# Differential Expression of Cytotoxic-Associated Markers in Circulating CD4^+^CD28^−^ T Cells and NK Cells in Rheumatoid Arthritis

**DOI:** 10.3390/ijms27198532

**Published:** 2026-09-24

**Authors:** Julio Sesma-Soto, Mariana Patlán, Rashidi Springall, Araceli Páez, Felipe Massó, Luis H. Silveira, Luis M. Amezcua-Guerra

**Affiliations:** 1 Department of Immunology, Instituto Nacional de Cardiología Ignacio Chávez, Mexico City 14080, Mexico; juliosess187@gmail.com (J.S.-S.); raspringall@yahoo.com (R.S.); 2 Basic Research Subdirectorate, Instituto Nacional de Cardiología Ignacio Chávez, Mexico City 14080, Mexico; mariana.patlan@cardiologia.org.mx; 3 UNAM/INC Translational Research Unit, Instituto Nacional de Cardiología Ignacio Chávez, Mexico City 14080, Mexico; arapaez@yahoo.com (A.P.); f_masso@yahoo.com (F.M.); 4 Department of Rheumatology, Instituto Nacional de Cardiología Ignacio Chávez, Mexico City 14080, Mexico; luis_hsil@yahoo.com; 5 Health Care Department, Universidad Autónoma Metropolitana–Xochimilco, Mexico City 04960, Mexico; 6 School of Medicine, Universidad La Salle, Mexico City 06140, Mexico

**Keywords:** rheumatoid arthritis, CD4^+^CD28^−^ cells, immune senescence, cytotoxicity, Fas ligand, granzyme B, perforin

## Abstract

Rheumatoid arthritis (RA) involves alterations in cytotoxic lymphoid differentiation, yet the precise phenotypic remodeling of circulating helper T cells and natural killer (NK) cells remains incompletely understood. We evaluated circulating CD4^+^CD28^+^ and CD4^+^CD28^−^ T-cell subsets and NKG2A^+^CD56^+^ NK cells, alongside granule-mediated cytotoxic markers (granzyme B and perforin) and the surface-bound death receptor Fas ligand (FasL), in 28 female patients with RA and 12 age-matched healthy female controls using multiparameter flow cytometry. CD4^+^CD28^−^ cells were increased in RA compared with controls (16.6% versus 6.5% of CD4^+^ cells). Within CD4^+^CD28^−^ cells, granzyme B positivity was markedly lower in RA (5.0% versus 62.0%), whereas FasL expression was higher (40.3% versus 22.5%). RA patients also exhibited a higher proportion of circulating NKG2A^+^CD56^+^ NK cells (6.1% versus 2.7% of PBMCs) with substantially lower granzyme B expression (8.4% versus 55.6%). Higher disease activity (DAS28-CRP) correlated positively with total NKG2A^+^CD56^+^ NK cell frequencies and intracellular perforin expression in both CD4^+^CD28^+^ T cells and NKG2A^+^CD56^+^ NK cells, as well as lower FasL expression in CD4^+^CD28^−^ cells. These findings support a disease-associated phenotypic remodeling of circulating cytotoxic markers in RA, characterized by shifted granzyme B and FasL expressions in CD4^+^CD28^−^ T cells and NKG2A^+^CD56^+^ NK cells, although whether these profiles translate into altered cytotoxic kinetics remains to be functionally determined.

## 1. Introduction

Rheumatoid arthritis (RA) is a chronic systemic autoimmune disease characterized by sustained immune activation leading to synovial inflammation, progressive joint destruction, and a broad spectrum of extra-articular manifestations [1]. The disease arises from a complex interplay between genetic susceptibility, environmental triggers, and the loss of immune tolerance—a process frequently accompanied by the emergence of disease-associated autoantibodies years before clinical onset [2]. Prominent among these are autoantibodies targeting post-translationally modified proteins, such as anti-citrullinated protein antibodies (ACPAs) and anti-carbamylated protein (anti-CarP) antibodies. Central to RA pathogenesis is the aberrant activation of adaptive immune responses, particularly CD4^+^ T cells, which orchestrate inflammatory cascades through the production of cytokines such as interferon-gamma (IFN-γ), interleukin-17 (IL-17), and IL-6, as well as through dynamic crosstalk with myeloid and stromal cells [3].

Among the T-cell abnormalities described in RA, the expansion of circulating CD4^+^ T cells lacking expression of the co-stimulatory molecule CD28 (CD4^+^CD28^−^, also referred to as CD4^+^CD28^null^ cells) has consistently been associated with disease severity [4,5]. However, CD4^+^CD28^−^ cells do not constitute a discrete immunologic lineage; rather, they represent a phenotypically defined and heterogeneous compartment that emerges under chronic antigenic stimulation. This compartment encompasses cells at different stages of differentiation and activation, exhibiting variable degrees of senescence and exhaustion, cytotoxic potential, and the acquisition of natural killer (NK)-associated features. Although CD4^+^CD28^−^ cells express cytotoxic molecules such as perforin and granzymes and have been implicated in accelerated atherosclerosis and increased cardiovascular risk in RA, their biological and functional heterogeneity remains incompletely understood [4,5].

Crucially, this pathogenic acquisition of a cytotoxic profile by CD4^+^ T cells mirrors phenotypic shifts observed within innate lymphoid populations in RA, particularly NK cells. Current evidence shows that synovial NK cells may aggravate arthritis through the production of inflammatory mediators, such as granulocyte–macrophage colony-stimulating factor (GM-CSF) and the receptor activator of nuclear factor-kappa B ligand (RANKL), thereby priming effector myeloid cells. In contrast, peripheral NK cells may exert a protective role in RA by virtue of IFN-γ production and cytotoxicity against arthritogenic immune cells [6]. Nevertheless, growing evidence suggests that peripheral NK cell compartments undergo profound functional changes during active disease; specifically, lower circulating NK cell frequencies and diminished activity have been directly linked to higher disease activity and systemic inflammation [7]. Furthermore, recent phenotypic profiling shows that the downregulation of granule-associated cytotoxic mediators, such as perforin and granzyme B, on peripheral NK cells correlates with severe clinical phenotypes, including bone deformity and erosive disease [8,9].

Paradoxically, while several studies have reported increased expression of cytotoxicity-associated molecules within CD4^+^CD28^−^ T cells, the comprehensive phenotypic distribution of these markers in RA has not been fully characterized [5]. In particular, the expression patterns of molecules associated with granule-mediated cytotoxicity, including perforin and granzyme B, and death receptor-mediated pathways, such as Fas ligand (FasL), remain insufficiently explored in this population [5]. Importantly, the expression of these molecules should not be interpreted as direct evidence of cytotoxic function, as they may reflect distinct activation states or functional exhaustion and do not necessarily predict definitive target-cell killing. Nevertheless, a comprehensive characterization of their expression profile may provide insights into the phenotypic features of the CD4^+^CD28^−^ compartment and generate hypotheses regarding its potential contribution to disease pathogenesis.

Overall, these data suggest a widespread, systemic dysregulation of cytotoxic pathways spanning across both innate and adaptive cellular lineages in RA [10]. In the present study, we performed a comparative phenotypic characterization of circulating CD4^+^CD28^+^ and CD4^+^CD28^−^ T-cell subsets and NKG2A^+^CD56^+^ NK cells in patients with RA and healthy controls by assessing the expression of granzyme B, perforin, and FasL using multiparameter flow cytometry [11]. We also explored the association between these coordinated phenotypic features and clinical disease activity.

## 2. Results

### 2.1. Study Population

A total of 28 female patients with RA and 12 age- and sex-matched healthy controls were included in this study (Table 1). The median age was comparable between the two groups: 51 years (interquartile range [IQR], 40–55) for patients with RA versus 47 years (IQR, 38–51) for healthy controls (*p* = 0.360). Among the RA cohort, the median disease duration was 6.5 years (IQR, 4.2–14.0). Regarding comorbidities, systemic hypertension and type 2 diabetes mellitus were each present in 25% of the patients, while Sjögren’s disease was present in 17%.

In terms of inflammatory markers, RA patients exhibited a median high-sensitivity C-reactive protein (hsCRP) level of 4.93 mg/L (IQR, 1.78–10.0) and a median erythrocyte sedimentation rate (ESR) of 8.5 mm/h (IQR, 4.0–23.7). Rheumatoid factor (RF) was detected in 75% of patients (median, 119 IU/mL [IQR, 40–319]), and ACPAs were detected in 64% (median, 250 IU/mL [IQR, 67–1038]). The study population received heterogeneous immunomodulatory therapies, which included conventional synthetic disease-modifying antirheumatic drugs (csDMARDs)—predominantly methotrexate (78%) and sulfasalazine (60%)—as well as glucocorticoids (14%), biologic DMARDs (bDMARDs; 10%), and targeted synthetic DMARDs (tsDMARDs; 17%) (Table 1).

### 2.2. Altered Frequencies of Circulating CD4^+^ T-Cell Subsets and NKG2A^+^CD56^+^ NK Cells in RA

The proportion of total T cells within the peripheral blood mononuclear cell (PBMC) fraction was comparable between RA patients and healthy controls (24.8% versus 27.9%; *p* = 0.615). Similarly, no significant differences were observed in the percentage of CD4^+^ T cells within the total T-cell compartment between the two groups (41.6% versus 37.4%; *p* = 0.647). In contrast, the frequency of CD4^+^CD28^+^ T cells was significantly decreased in the RA cohort compared to healthy controls (83.3% versus 93.4%; *p* = 0.002) (Table 2).

Concurrently, the CD4^+^CD28^−^ T-cell subset was significantly expanded in patients with RA (16.6% versus 6.5%; *p* = 0.002). Regarding NKG2A^+^CD56^+^ NK cells, their percentage relative to total PBMCs was significantly higher in the RA group than in the control group (6.1% versus 2.7%; *p* = 0.023) (Table 2).

### 2.3. Differential Expression of Cytotoxicity-Associated Markers in CD4^+^ T-Cell Subsets and NKG2A^+^CD56^+^ NK Cells

In healthy individuals, CD4^+^CD28^−^ T cells represented a median of 6.5% of total circulating CD4^+^ cells. Although this subset accounted for a relatively small proportion of the circulating CD4^+^ pool, it exhibited significantly higher proportions of granzyme B-positive, perforin-positive, and FasL-positive cells than its CD4^+^CD28^+^ counterpart. Specifically, granzyme B positivity was observed in 62.0% of CD4^+^CD28^−^ cells compared with only 5.0% of CD4^+^CD28^+^ cells (*p* = 0.012). Likewise, perforin-positive cells were markedly more frequent within the CD4^+^CD28^−^ subset than in the CD4^+^CD28^+^ population (6.6% versus 0.3%; *p* = 0.003), as were FasL-positive cells (22.5% versus 12.3%; *p* = 0.012) (Table 2).

Among RA patients, the CD4^+^CD28^−^ subset was expanded, accounting for 16.6% of total circulating CD4^+^ T cells. Similar to the pattern observed in healthy controls, RA CD4^+^CD28^−^ T cells contained significantly higher proportions of granzyme B-positive (5.0% versus 0.8%; *p* = 0.001), perforin-positive (6.7% versus 0.7%; *p* = 0.002), and FasL-positive cells (40.3% versus 10.8%; *p* < 0.001) than paired CD4^+^CD28^+^ cells (Table 2).

Direct comparison of the corresponding T-cell subsets between the RA and control groups revealed distinct expression patterns. Within the CD4^+^CD28^+^ compartment, RA patients exhibited a lower proportion of granzyme B-positive cells (0.8% versus 5.0%; *p* = 0.001) and a slightly higher proportion of perforin-positive cells (0.7% versus 0.3%; *p* = 0.042) than healthy controls, whereas FasL expression did not differ significantly between groups. Within the CD4^+^CD28^−^ compartment, granzyme B positivity was markedly lower in the RA cohort than in healthy controls (5.0% versus 62.0%; *p* < 0.001), whereas perforin positivity remained comparable (6.7% versus 6.6%; *p* = 0.872). Conversely, the proportion of FasL-positive CD4^+^CD28^−^ cells was significantly higher in RA patients than in healthy controls (40.3% versus 22.5%; *p* = 0.048).

Regarding the NKG2A^+^CD56^+^ NK-cell compartment, RA patients exhibited a profoundly lower expression of granzyme B compared to healthy controls (8.4% versus 55.6%; *p* < 0.001). In contrast, the percentages of NKG2A^+^CD56^+^ NK cells expressing perforin (5.0% versus 6.7%; *p* = 0.790) and FasL (91.8% versus 95.9%; *p* = 0.497) were similar between the RA and healthy control groups (Table 2).

### 2.4. Exploratory Analysis According to Disease Activity

When stratified by the Disease Activity Score for 28 joints using the C-reactive protein (DAS28-CRP) index, patients with lower disease activity (median DAS28-CRP = 1.9 [range, 1.1–2.8]) exhibited a significantly higher proportion of granzyme B-positive CD4^+^CD28^+^ T cells (1.2% versus 0.2%; *p* = 0.021) than those with higher disease activity (median DAS28-CRP = 3.9 [range, 3.3–5.3]). Conversely, patients with higher disease activity exhibited greater proportions of perforin-positive cells within both the CD4^+^CD28^+^ subset (6.0% versus 0.5%; *p* = 0.038) and the CD4^+^CD28^−^ subset (18.3% versus 3.9%; *p* = 0.018). Furthermore, the frequency of FasL-positive CD4^+^CD28^−^ cells was significantly lower in the higher disease activity group compared to the lower disease activity group (21.4% versus 44.7%; *p* = 0.043) (Table 3).

Regarding the NKG2A^+^CD56^+^ NK-cell compartment, no significant differences were observed in either the total cell frequency or the percentages of cells expressing granzyme B, perforin, or FasL when comparing patients with low and high disease activity (Table 3).

To further dissect the continuous relationship between systemic inflammatory burden and cytotoxic signatures, a Spearman rank correlation analysis was executed across the entire RA cohort (Table 4). Clinical disease activity (DAS28-CRP) exhibited significant, positive correlations with intracellular perforin expression within both conventional CD4^+^CD28^+^ T cells (ρ = 0.406, 95% CI: 0.027 to 0.683; *p* = 0.031) and NKG2A^+^CD56^+^ NK cells (ρ = 0.439, 95% CI: 0.067 to 0.704; *p* = 0.019). Higher DAS28-CRP scores also correlated with an increased total frequency of circulating NKG2A^+^CD56^+^ NK cells (ρ = 0.385, 95% CI: 0.002 to 0.669; *p* = 0.043). No significant correlations were observed between the DAS28-CRP and the remaining T-cell or NK-cell immunophenotypes, including their respective expression of granzyme B or FasL.

After Benjamini–Hochberg adjustment within each predefined cell population, the correlation between DAS28-CRP and perforin expression in CD4^+^CD28^+^ T cells remained statistically significant (FDR-adjusted *p* = 0.047). The association between DAS28-CRP and perforin expression in NKG2A^+^CD56^+^ NK cells remained close to, but did not reach, the prespecified FDR threshold (FDR-adjusted *p* = 0.057).

## 3. Discussion

The present study demonstrates that RA is associated with an increased frequency of circulating CD4^+^CD28^−^ cells and NKG2A^+^CD56^+^ NK cells, concomitantly accompanied by a profoundly altered expression pattern of cytotoxicity-associated markers within both cellular compartments. Compared with healthy controls, RA CD4^+^CD28^−^ T cells and peripheral NKG2A^+^CD56^+^ NK cells exhibited markedly lower intracellular granzyme B expression. Conversely, surface expression of FasL was significantly upregulated within the CD4^+^CD28^−^ T-cell pool, whereas perforin expression remained largely preserved across both lineages. These findings identify a distinct, parallel phenotypic remodeling of circulating innate and adaptive cytotoxic populations in RA, while exploratory analyses further suggest that these profiles undergo coordinated shifts according to clinical disease activity [12].

CD4^+^CD28^−^ cells have traditionally been regarded as terminally differentiated effector-memory T cells that emerge following chronic antigenic stimulation, including persistent viral infections and autoimmune responses [13,14,15]. These cells are characteristically enriched with cytotoxic mediators and frequently express perforin, granzymes, FasL, and several NK-associated receptors, supporting their recognized cytotoxic potential [16,17,18,19]. However, increasing evidence indicates that CD4^+^CD28^−^ cells represent a biologically heterogeneous phenotypic compartment rather than a discrete immunological lineage, encompassing cells with variable differentiation, activation, senescence, and functional states [20]. Because our flow cytometry panel did not incorporate additional specific markers—such as CD57 or KLRG1 for senescence, PD-1 or TIM-3 for cellular exhaustion, or HLA-DR for current activation status—we cannot definitively dissect the precise intra-compartmental dynamics of these cells in our cohort. The observed expansion and altered cytotoxic cargo could reflect an accumulation of terminally differentiated effector cells, a state of chronic activation, or varying degrees of functional exhaustion induced by the systemic inflammatory environment of RA.

Consistent with previous reports, CD4^+^CD28^−^ cells displayed higher baseline expression of cytotoxicity-associated markers than their conventional CD4^+^CD28^+^ counterparts in both healthy individuals and patients with RA, supporting the concept that the loss of CD28 is inextricably linked to the acquisition of cytotoxic properties [16,17,18,19]. However, a direct comparison between RA patients and healthy controls revealed a distinctive and paradoxical expression pattern characterized by reduced granzyme B and increased FasL expression, while perforin levels remained relatively stable. Although these findings indicate that RA is associated with an altered cytotoxic phenotype within the CD4^+^CD28^−^ compartment, they should not be interpreted as definitive evidence of preferential utilization of one cytotoxic pathway over another, as no direct functional killing assays were performed.

Crucially, this phenotypic remodeling within the adaptive arm is symmetrically mirrored in the innate compartment. Our data revealed that while the total proportion of circulating NKG2A^+^CD56^+^ NK cells was elevated in RA patients, these cells exhibited a profoundly blunted expression of granzyme B compared to healthy controls, while perforin and FasL remained unchanged [8,9]. This parallel downregulation of granzyme B in both CD4^+^CD28^−^ T cells and NKG2A^+^CD56^+^ NK cells suggests a shared, systemic dysregulation of the granule-mediated cytolytic machinery [10]. It is important to underscore that our study specifically analyzed the NKG2A^+^CD56^+^ NK-cell subset. While total circulating NK cells are frequently reported to be decreased in active RA and inversely correlated with systemic inflammation, our rigorous multiple testing correction (FDR) demonstrated no robust statistical relationship between disease activity and cellular frequency in this cohort. This highlights that apparent discrepancies with previous literature may stem from both statistical constraints in small cohorts and crucial differences in phenotypic definitions, as evaluating a specific receptor-bearing subset (NKG2A^+^) captures a distinct immunological dynamic compared to analyzing the global circulating NK-cell pool.

Our exploratory analyses also suggested that expression of cytotoxicity-associated markers may vary according to inflammatory activity. Patients with higher disease activity tended to exhibit differences in perforin and FasL expression compared with those with lower disease activity, suggesting that the relative use of granule-mediated versus death-receptor pathways may shift with inflammatory burden [6,8]. However, these subgroup analyses were based on a limited number of patients and should therefore be considered hypothesis-generating.

Several non-mutually exclusive mechanisms could account for these systemic phenotypic alterations. First, the chronic, unremitting antigenic and inflammatory stimulation characteristic of RA could trigger repeated, low-level degranulation of cytotoxic granule stores in the periphery. This persistent release could result in the apparent exhaustion and progressive depletion of intracellular granzyme B pools despite a preserved cellular capacity for perforin expression or independent replenishment [21]. This phenomenon aligns with established models of cellular exhaustion in chronic inflammatory diseases, where persistent activation leads to a selective loss of effector molecule storage [22].

Second, the structural dichotomy observed between the peripheral blood and the target tissue must be considered. In active RA, peripheral NK cells and highly differentiated T cells undergo chemokinetic reprogramming, prompting the preferential migration (homing) of highly cytolytic, granzyme B-rich effector cells into the inflamed synovial fluid, where they actively aggravate joint destruction by priming myeloid cells and fibroblasts [6]. Consequently, this selective tissue recruitment may leave a signature footprint in the circulation, characterized by an overrepresentation of granzyme B-depleted and functionally exhausted innate and adaptive cells.

Third, local or systemic inflammatory cues—specifically cytokine milieus dominated by tumor necrosis factor (TNF) and IL-6—may transcriptionally or post-translationally influence these effector pathways, selectively upregulating FasL as an alternative, non-secretory cytotoxic mechanism [14,21]. Functionally, a FasL-biased phenotype in circulating CD4^+^CD28^−^ T cells could promote the apoptosis of Fas-expressing synovial or endothelial targets upon contact, directly linking the expansion of this compartment to the accelerated atherosclerosis and tissue injury observed in these patients [14,23]. However, our present data do not allow definitive discrimination between these intracellular possibilities, nor do they establish whether these observed shifts translate into altered net cytotoxic function in vivo.

Regarding perforin expression, our unadjusted analyses initially hinted at potential trends with clinical scores. However, after adjusting for multiple statistical tests, no robust continuous correlations could be confirmed between systemic inflammatory burden and intracellular perforin cargo. Mechanistically, while chronic stimulation leads to the functional exhaustion and depletion of granzyme B stores, the transcriptional and translational machinery of perforin appears to be actively upregulated by the high circulating levels of pro-inflammatory cytokines, such as IL-6 and TNF [8]. This distinct regulatory dichotomy between the two granule-mediated components suggests that perforin synthesis remains tightly coupled to the kinetic velocity of current systemic inflammation, whereas granzyme B kinetics are dictated by chronic, long-term cellular exhaustion. In support of this assertion, while active systemic disease triggers the numeric expansion or mobilization of NK cells into the peripheral blood, these newly recruited innate cells are phenotypically dysfunctional, carrying a severely blunted granzyme B cargo, yet their perforin machinery remains intact. This finding expands upon the paradigm of innate immune dysregulation in RA; severe clinical phenotypes and high inflammatory states not only alter the absolute distribution of circulating lymphoid lineages but also systematically reshape their intracellular effector profiles, rendering them non-secretory or functionally polarized [7].

The distinctive phenotypic profiles observed in circulating CD4^+^CD28^−^ T cells and NKG2A^+^CD56^+^ NK cells may nevertheless hold substantial clinical and therapeutic relevance. The expansion of these cytolytic compartments has previously been associated with severe joint damage and extra-articular manifestations [23,24]. However, a major challenge in interpreting these findings is the confounding impact of the heterogeneous immunomodulatory therapies received by our cohort. Conventional synthetic DMARDs like methotrexate and sulfasalazine, which were taken by most of our patients, can exert profound antiproliferative and anti-inflammatory effects on peripheral lymphocytes. Similarly, therapies targeting T-cell co-stimulation, such as abatacept, have been shown to progressively reduce circulating CD4^+^CD28^−^ T-cell frequencies after prolonged treatment [14,25,26]. Whether current bDMARDs or tsDMARDs also modify or restore the intracellular expression of granzyme B and other cytotoxicity-associated molecules within these compartments remains an important open question for future investigation.

This study has several limitations. First, its cross-sectional design precludes the assessment of temporal kinetics or definitive causal relationships. Second, the relatively small sample size, particularly for the disease activity stratification, limits statistical power and requires cautious interpretation of the exploratory findings. Third, the RA cohort was therapeutically heterogeneous, and the potential isolated influence of glucocorticoids and various DMARDs on cytotoxic marker expression could not be statistically evaluated. Because these potent immunomodulatory agents can themselves systematically reshape peripheral lymphocyte phenotypes and alter the expression of granule-associated proteins, the present cross-sectional design cannot definitively determine the extent to which the observed phenotype is driven by the primary pathophysiological mechanisms of RA versus the pharmacological footprint of ongoing therapies. Fourth, our analysis relied exclusively on immunophenotypic characterization by flow cytometry and did not include functional assays to assess active degranulation, apoptosis induction, or target-cell killing. Fifth, because this study exclusively enrolled female participants to eliminate sex-associated biological confounding on immune cell counts and clinical activity scores, our findings represent a sex-specific immunophenotypic profile. Finally, circulating populations were defined according to cell-surface and intracellular markers, and they should not be interpreted as representing perfectly homogeneous cellular lineages.

## 4. Materials and Methods

### 4.1. Study Design and Participant Selection

This observational, cross-sectional study enrolled consecutive adult patients with RA attending a tertiary referral center embedded within a university-affiliated academic hospital. All patients fulfilled the 2010 American College of Rheumatology/European Alliance of Associations for Rheumatology (ACR/EULAR) classification criteria for RA [11]. To minimize potential confounding biases on disease activity indices and peripheral immune cell counts related to biological sex, the study design exclusively included female participants. Individuals with an ongoing pregnancy, active infections, malignancies, chronic viral infections (such as human immunodeficiency virus or viral hepatitis), or concomitant systemic autoimmune diseases (with the exception of Sjögren’s disease) were excluded. Age-matched healthy female volunteers without underlying inflammatory or autoimmune conditions were recruited to serve as the control group.

### 4.2. Clinical and Laboratory Assessment

Demographic and clinical variables—including age, disease duration, and major comorbidities (e.g., type 2 diabetes mellitus, systemic hypertension, and Sjögren’s disease)—were systematically recorded. Current pharmacotherapy with glucocorticoids, csDMARDs, bDMARDs, and sDMARDs was documented.

Routine laboratory evaluations included complete blood count, ESR, and hsCRP levels. RF and ACPA were quantified using nephelometry and enzyme-linked immunosorbent assays (ELISA), respectively, according to the manufacturers’ protocols. Clinical disease activity was assessed utilizing the validated three-variable 28-joint Disease Activity Score calculated with hsCRP (DAS28-CRP; https://www.das-score.nl/das28/DAScalculators/dasculators.html [accessed between June 2023 and October 2023]). Stratified exploratory analyses were subsequently performed based on established clinical disease activity categories classified dichotomously as follows: low disease activity was defined as a DAS28-CRP score <3.0 points, whereas high disease activity was defined as a DAS28-CRP score ≥3.0 points.

### 4.3. Ethical Considerations

All participants provided written informed consent prior to enrollment. The study protocol was approved by the Institutional Ethics and Research Committees of the Instituto Nacional de Cardiología Ignacio Chávez (protocol approval number: 21-1219) and was conducted in strict accordance with the ethical principles outlined in the Declaration of Helsinki.

### 4.4. PBMC Isolation and Flow Cytometry

#### 4.4.1. Cell Isolation and Immunophenotyping Staining

Peripheral blood samples were collected in tubes containing ethylenediaminetetraacetic acid (EDTA) as an anticoagulant. PBMCs were immediately isolated via density-gradient centrifugation using Histopaque-1077 (Sigma-Aldrich, St. Louis, MO, USA). Isolated PBMCs were washed twice with phosphate-buffered saline (PBS), and cell counts were determined using an automated cell counter (Corning, Amsterdam, The Netherlands). Cell suspensions were subsequently adjusted to a final concentration of 5 × 10^5^ cells per staining tube.

Surface immunophenotyping was performed using a panel of previously titrated fluorochrome-conjugated monoclonal antibodies directed against CD4 (BUV737, BD Horizon, Singapore, Cat. No. 612748), CD28 (PE, Tonbo Biosciences, San Diego, CA, USA, Cat. No. 50-0286-T025), CD56 (FITC, BD Biosciences, Singapore, Cat. No. 555516), FasL/CD178 (BV421, BD OptiBuild, BD Biosciences, Cat. No. 744097), and NKG2 (BV605, BD Biosciences, Cat. No. 747921). Following surface antigen staining, cells were fixed and permeabilized using the IC Fixation Buffer Kit (Invitrogen, Carlsbad, CA, USA, Cat. No. 00-8222-49) according to the manufacturer’s instructions. Intracellular staining was subsequently performed using monoclonal antibodies against perforin (AF488, BD Pharmingen, BD Biosciences, Cat. No. 563764) and granzyme B (AF647, BD Pharmingen, Cat. No. 560212). For instrument setup and data validation, fluorescence minus one (FMO) controls and unstained cells were acquired during each experimental run, while single-stained cells were utilized to generate automated fluorescence compensation matrices.

#### 4.4.2. Gating Strategy and Cytotoxic Marker Quantification

Flow cytometric data acquisition was performed, and a sequential gating strategy was established to delineate both adaptive and innate cell populations. Initially, lymphocytes were identified based on their characteristic forward-scatter (FSC) and side-scatter (SSC) properties. Cell doublets were rigorously excluded by analyzing forward-scatter height versus forward-scatter area (FSC-H versus FSC-A) profiles to ensure the analysis of single cells.

For the T-cell compartment, CD4^+^CD56^−^ cells were selected from the singlet gate, defined by FSC-A vs. FSC-H. Although a formal anti-CD3 marker was omitted in this panel, the combination of strict FSC/SSC lymphocyte gating (which excludes monocytes) and the intentional exclusion of CD56^+^ lineages effectively isolates conventional CD4^+^ T helper compartments in peripheral blood by removing CD4^+^ NK, CD8^+^, and NKT-like cells. This CD4^+^CD56^−^ population was then discriminated according to CD28 expression, defining the conventional CD4^+^CD28^+^ and the expanded CD4^+^CD28^−^ subsets. Concurrently, the NK-cell compartment was defined within the same singlet gate as NKG2A^+^CD56^+^ cells. This strategy was used to identify a defined NK-cell population and was not intended to represent the entire circulating NK-cell compartment. Finally, the frequencies of the cytotoxicity-associated markers—granzyme B, perforin, and FasL—were quantified within each specific CD4^+^ T-cell subset and the NKG2A^+^CD56^+^ NK-cell compartment using specific boundary gates determined by their respective unstained and fluorescence minus one (FMO) controls. Representative gating plots are shown in Figure 1.

Flow cytometric acquisition was performed using a BD FACSAria Fusion flow cytometer (BD Biosciences, San Jose, CA, USA); the data were collected with FACSDiva software v.9.7 (BD Biosciences, San Jose, CA, USA) and analyzed using FlowJo software v.10.10.1 (BD Biosciences).

### 4.5. Statistical Analysis

The normality of continuous variables was formally assessed using the Shapiro–Wilk test. Continuous data are presented as medians and interquartile ranges (IQRs) or ranges (minimum to maximum values), whereas categorical variables are expressed as absolute frequencies and percentages. Because most variables were not normally distributed, between-group comparisons were performed using nonparametric tests.

The Mann–Whitney U test was used for comparisons between two independent groups, whereas the Kruskal–Wallis test followed by Dunn’s multiple-comparison correction was applied when more than two groups were analyzed. Categorical variables were compared using Fisher’s exact test. The association between numerical variables was assessed using the Spearman rank correlation coefficient (ρ) with 95% confidence intervals (CIs). To account for multiple comparisons, the false discovery rate (FDR) was controlled using the Benjamini–Hochberg procedure within each predefined cell population.

All statistical evaluations were two-sided, and a *p*-value < 0.05 was considered to indicate statistical significance. All computational data processing and statistical analyses were performed using GraphPad Prism software version 11.0.2 (GraphPad Software, La Jolla, CA, USA).

## 5. Conclusions

In conclusion, RA is associated with a distinct, parallel remodeling of circulating immune cell populations, characterized by the expansion of both CD4^+^CD28^−^ T cells and NKG2A^+^CD56^+^ NK cells. This systemic alteration is marked by a selective downregulation of intracellular granzyme B alongside preserved perforin expression across both adaptive and innate lineages, concurrently coupled with a robust upregulation of surface FasL within the CD4^+^CD28^−^ T-cell compartment. These findings suggest a disease-associated phenotypic divergence between internal granule components and surface death-receptor ligands in the circulation. Because functional cytolytic assays were not performed, these patterns reflect structural cellular remodeling rather than a definitive shift in secretory or killing capabilities. Future functional assays are warranted to fully elucidate the translational implications of this cytotoxic dysregulation in the pathogenesis of RA.

## Figures and Tables

**Figure 1:**
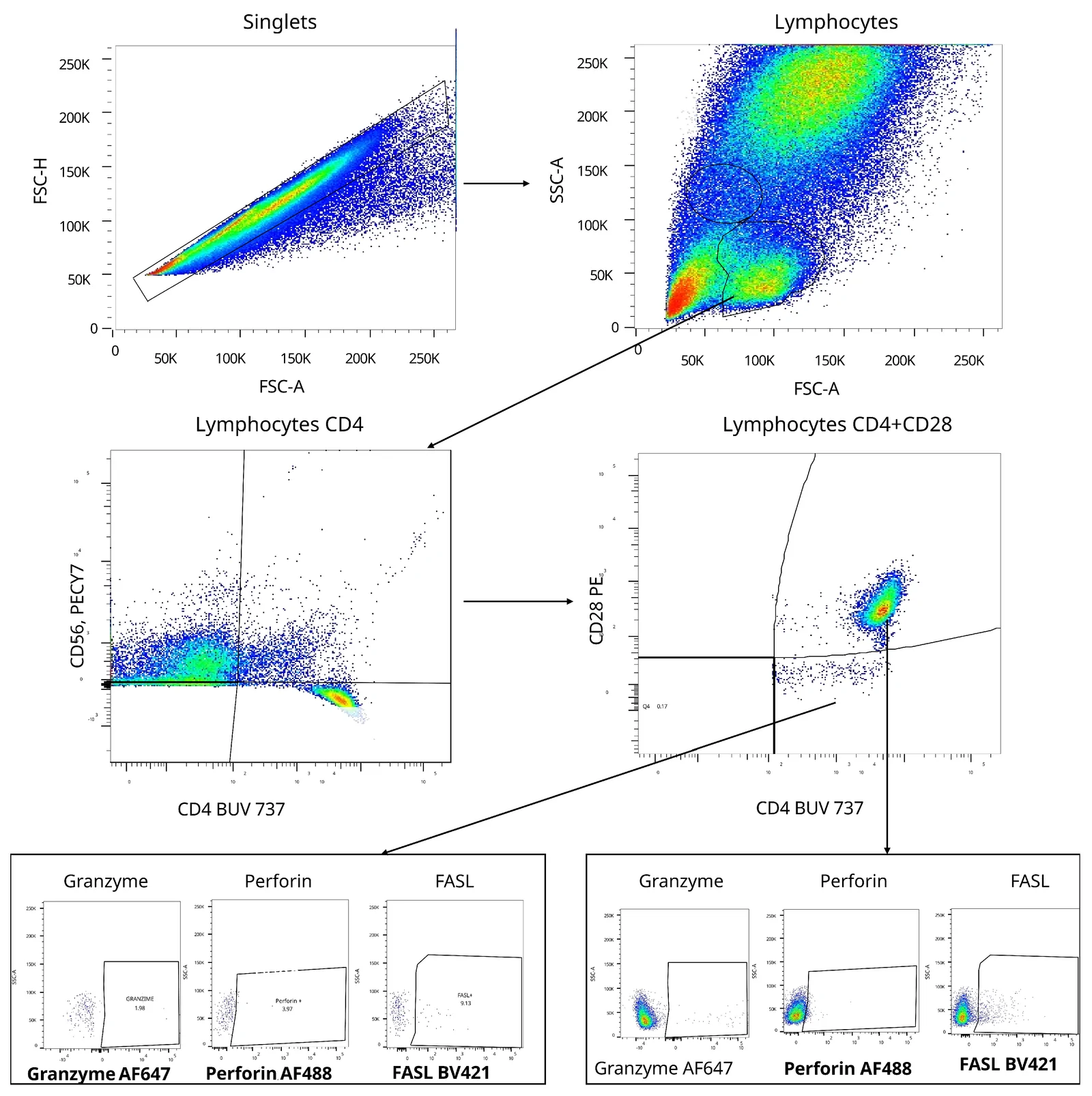

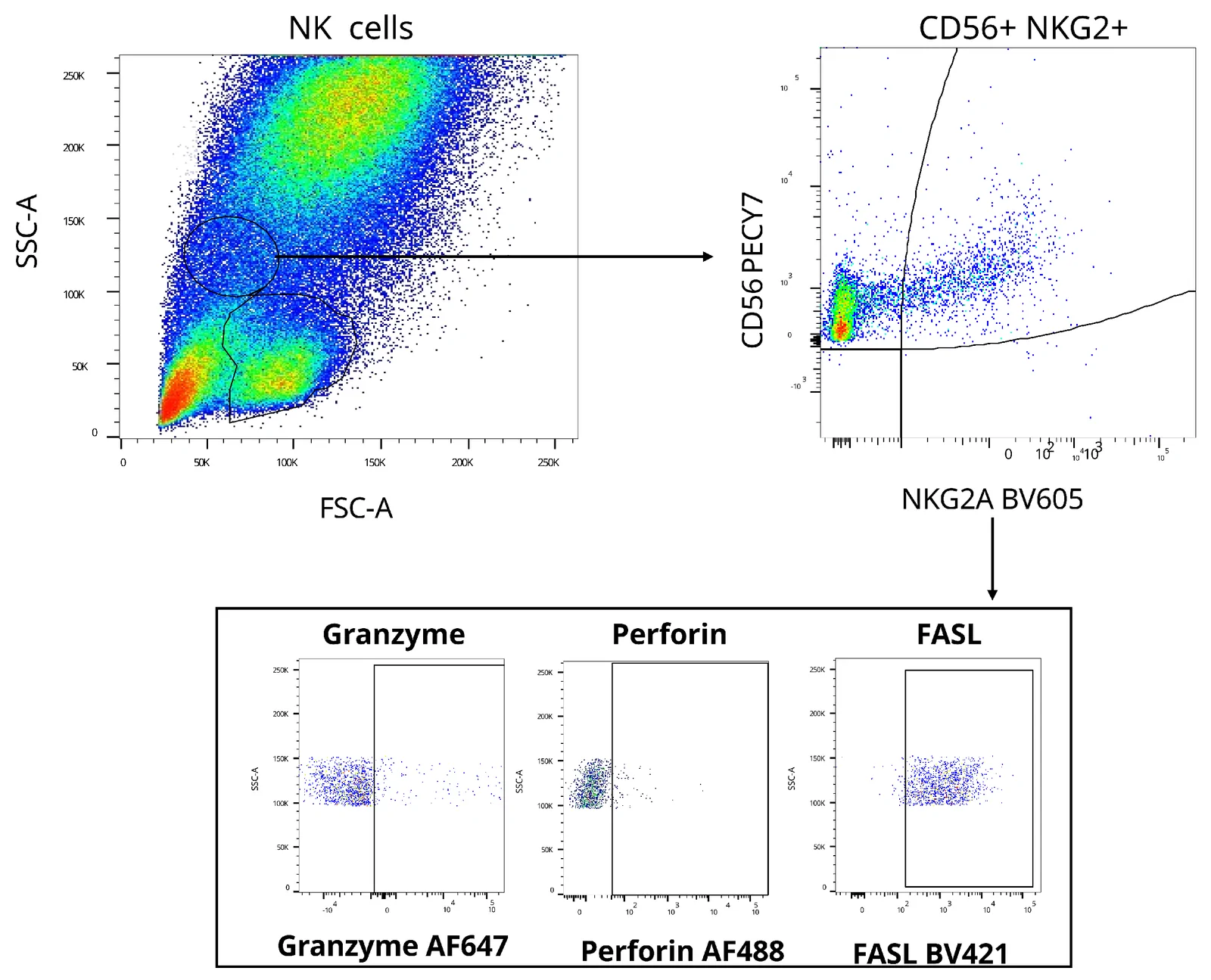
Flow cytometric sequential gating strategy for the immunophenotypic identification and cytotoxic characterization of circulating CD4^+^ T-cell subsets and natural killer (NK) cells. CD4^+^ T-cell compartment: Representative density plot demonstrating doublet exclusion to select single cells (singlets) based on forward-scatter height versus area (FSC-H vs. FSC-A). Morphological identification of the lymphocyte population according to size and internal granularity using side-scatter area versus forward-scatter area (SSC-A vs. FSC-A). Gating of CD4^+^CD56^−^ helper T lymphocytes, intentionally excluding CD56^+^ lineages. Representative quadrant gates discriminating the CD4^+^CD28^−^ subset (lower-right) and the conventional CD4^+^CD28^+^ population (upper-right) within the pre-gated CD4^+^ T-cell pool are shown. Representative dot plots evaluate the expression percentages of Fas ligand (FasL), granzyme B, and perforin within the isolated CD4^+^CD28^+^ and CD4^+^CD28^−^ lymphocyte subsets, respectively. NK-cell compartment: Identification of the innate NK cell population within the morphological singlet gate, defined phenotypically as NKG2A^+^CD56^+^ cells. Representative plots illustrate the quantification of intracellular granule-associated cytotoxic mediators, specifically granzyme B (AF647) and perforin (AF488), evaluated against side-scatter characteristics (SSC vs. fluorochrome). Representative plots illustrating the surface expression quantification of the death receptor ligand FasL (BV421) against side-scatter characteristics (SSC vs. FasL) are also shown. Boundary gates for all cytotoxic markers were strictly determined utilizing respective unstained and fluorescence minus one (FMO) controls.

**Table 1:** Demographic and clinical data from study participants.

Variable	Rheumatoid Arthritis (*n* = 28)	Healthy Controls (*n* = 12)	*p*-Value
Age, years	51 (40–55)	47 (38–51)	0.360
Female, *n* (%)	28 (100)	12 (100)	>0.999
Disease duration, years	6.5 (4.2–14.0)	-	-
Diabetes mellitus, *n* (%)	7 (25)	0	0.081
Hypertension, *n* (%)	7 (25)	1 (8.3)	0.395
Sjögren’s disease, *n* (%)	5 (17)	0	0.298
hsCRP, mg/L	4.93 (1.78–10.04)	-	-
ESR, mm/h	8.5 (4.0–23.7)	-	-
Rheumatoid factor +, *n* (%)	21 (75)	-	-
Rheumatoid factor, IU/mL	119 (40–319)	-	-
ACPAs +, *n* (%)	18 (64)	-	-
ACPAs, IU/mL	250 (67–1038)	-	-
Drug therapies, *n* (%)			
• Methotrexate	22 (78)	-	-
• Antimalarials	11 (39)	-	-
• Sulfasalazine	17 (60)	-	-
• Leflunomide	4 (14)	-	-
• Prednisone	4 (14)	-	-
• bDMARDs	3 (10)	-	-
• tsDMARDs	5 (17)	-	-

Data are presented as median (interquartile range) unless otherwise specified. Abbreviations: ACPAs, anti-citrullinated protein antibodies; bDMARDs, biological disease-modifying antirheumatic drugs; ESR, erythrocyte sedimentation rate; hsCRP, high-sensitivity C-reactive protein; tsDMARDs, targeted synthetic disease-modifying antirheumatic drugs.

**Table 2:** Phenotypic characterization in circulating CD4^+^ T-cell subsets and NKG2A^+^CD56^+^ NK cells in the study participants.

Cell Population/Marker	Rheumatoid Arthritis (*n* = 28)	Healthy Controls (*n* = 12)	*p*-Value
T cells, % of total PBMCs	24.8 (15.6–41.2)	27.9 (16.5–41.1)	0.615
CD4^+^ T cells; % of total T cells	41.6 (32.1–49.1)	37.4 (34.9–49.5)	0.647
CD4^+^CD28^+^ T cells, % of total CD4^+^ T cells	83.3 (52.2–90.6)	93.4 (88.3–95.8)	**0.002**
• Granzyme B^+^, %	0.8 (0.4–3.3)	5.0 (3.0–17.2)	**0.001**
• Perforin^+^, %	0.7 (0.2–3.0)	0.3 (0.1–0.6)	**0.042**
• FasL^+^, %	10.8 (5.6–31.1)	12.3 (9.4–14.0)	0.965
CD4^+^CD28^−^ T cells, % of total CD4^+^ T cells	16.6 (9.4–47.7)	6.5 (4.1–11.6)	**0.002**
• Granzyme B^+^, %	5.0 (1.8–16.0)	62.0 (28.3–75.5)	**<0.001**
• Perforin^+^, %	6.7 (2.0–15.2)	6.6 (1.0–13.8)	0.872
• FasL^+^, %	40.3 (20.3–62.2)	22.5 (12.4–36.6)	**0.048**
NKG2A^+^CD56^+^ NK cells, % of total PBMCs	6.1 (2.7–8.7)	2.7 (1.7–5.8)	**0.023**
• Granzyme B^+^, %	8.4 (5.0–25.1)	55.6 (41.7–69.9)	**<0.001**
• Perforin^+^, %	5.0 (2.9–7.7)	6.7 (1.8–12.5)	0.790
• FasL^+^, %	91.8 (78.7–98.2)	95.9 (91.5–97.8)	0.497

Data are presented as median (interquartile range). Significant *p*-values are indicated in bold. Abbreviations: NK, natural killer; PBMCs, peripheral blood mononuclear cells.

**Table 3:** Phenotypic characterization of circulating CD4^+^ T-cell subsets and NKG2A^+^CD56^+^ NK cells in RA patients stratified according to disease activity.

Cell Population/Marker	Low DAS28-CRP (*n* = 22)	High DAS28-CRP (*n* = 6)	*p*-Value
T cells, % of total PBMCs	24.8 (6.3–61.4)	25.4 (15.2–34.0)	0.695
CD4^+^ T cells; % of total T cells	37.9 (17.4–57.6)	47.8 (32.5–54.3)	0.262
CD4^+^CD28^+^ T cells, % of total CD4^+^ T cells	82.1 (19.7–96.3)	84.7 (47.5–90.6)	0.977
• Granzyme B^+^, %	1.2 (0.1–100)	0.2 (0.1–3.4)	**0.021**
• Perforin^+^, %	0.5 (0–13.6)	6.0 (0.3–87)	**0.038**
• FasL^+^, %	10.8 (1.3–71.1)	9.4 (4.9–63.1)	0.955
CD4^+^CD28^−^ T cells, % of total CD4^+^ T cells	17.8 (3.6–80.2)	15.2 (9.4–52.4)	0.955
• Granzyme B^+^, %	7.0 (0.5–75.1)	2.0 (0.4–7.8)	0.056
• Perforin^+^, %	3.9 (0.1–38.1)	18.3 (3.9–32.5)	**0.018**
• FasL^+^, %	44.7 (11.4–89.6)	21.4 (9.9–62.2)	**0.043**
NKG2A^+^CD56^+^ NK cells, % of total PBMCs	5.9 (0.8–25.4)	7.6 (3.5–14.1)	0.130
• Granzyme B^+^, %	8.3 (1.9–57.8)	8.9 (2.9–26.3)	0.779
• Perforin^+^, %	4.3 (0.2–40.8)	7.9 (2.5–20.1)	0.161
• FasL^+^, %	91.5 (9.6–99.7)	93.9 (87.4–98.3)	0.575

Data are presented as median (minimum-to-maximum range). Significant *p*-values are indicated in bold. Abbreviations: DAS28-CRP, Disease Activity Score for 28 joints using C-reactive protein. Low disease activity was defined as a DAS28-CRP score less than 3 points; high disease activity was defined as a DAS28-CRP score greater than or equal to 3 points.

**Table 4:** Spearman correlations between DAS28-CRP and circulating immunophenotypes.

Immunophenotype	ρ	95% CI	*p*-Value
CD4^+^ T cells, % of total T cells	0.113	−0.283 to 0.475	0.568
CD4^+^CD28^+^ T cells, % of total CD4^+^ T cells	0.090	−0.303 to 0.458	0.645
CD4^+^CD28^+^ Granzyme B^+^ (%)	−0.188	−0.533 to 0.210	0.337
CD4^+^CD28^+^ Perforin^+^ (%)	0.406	0.027 to 0.683	**0.031**
CD4^+^CD28^+^ FasL^+^ (%)	−0.039	−0.416 to 0.349	0.841
CD4^+^CD28^−^T cells, % of total CD4^+^ T cells	−0.087	−0.456 to 0.305	0.653
CD4^+^CD28^−^ Granzyme B^+^ (%)	−0.161	−0.512 to 0.237	0.413
CD4^+^CD28^−^ Perforin^+^ (%)	0.339	−0.050 to 0.639	0.077
CD4^+^CD28^−^ FasL^+^ (%)	−0.256	−0.582 to 0.141	0.189
NKG2A^+^CD56^+^ NK cells, % of total PBMCs	0.385	0.002 to 0.669	**0.043**
NKG2A^+^CD56^+^ NK cells, Granzyme B^+^ (%)	−0.209	−0.548 to 0.189	0.286
NKG2A^+^CD56^+^ NK cells, Perforin^+^ (%)	0.439	0.067 to 0.704	**0.019**
NKG2A^+^CD56^+^ NK cells, FasL^+^ (%)	0.081	−0.311 to 0.451	0.679

Spearman rank correlation coefficients (ρ) were calculated utilizing 28 paired observations for each clinical–immunophenotypic comparison. Significant *p*-values are indicated in bold. All tests were two-tailed. Abbreviations: DAS28-CRP, Disease Activity Score for 28 joints using C-reactive protein; CI, confidence interval; FasL, Fas ligand; NK, natural killer; PBMCs, peripheral blood mononuclear cells.

## Data Availability

The original contributions presented in this study are included in the article. Further inquiries can be directed to the corresponding author.
